# Psychotic relapse from COVID-19 pandemic: Clinical features

**DOI:** 10.1192/j.eurpsy.2021.1797

**Published:** 2021-08-13

**Authors:** L. Brahmi, H. Ben Ammar, E. Khelifa, G. Hamdi, R. Felhi, L. Mnif

**Affiliations:** Psychiatry Department, Razi hospital, manouba, Tunisia

**Keywords:** COVID-19, pandemic, psychosis, delusions

## Abstract

**Introduction:**

The COVID-19 pandemic affected today more than 76,000,000 worldwide, and more than half of humanity has been placed in quarantine. This pandemic affects mental health problems and influences the onset of symptoms.

**Objectives:**

The aim of this review is to analyze the impact of the COVID-19 pandemic on psychotic disorders and its interaction with the various risk factors.

**Methods:**

We undertook a review of the impact of COVID-19 pandemic on psychosis. We carried out a systematic review of electronic databases using the keywords “COVID-19”, “pandemics”, “psychotic disorders”, and “delusions”. Relevant literature was limited to articles conducted around the world and published between January and December 2020.

**Results:**

We identified ten papers addressing incident cases of psychosis relapse linked to coronavirus pandemic. In multiple cases, psychotic symptoms were characterized by delusional thoughts about being infected by the coronavirus. The limited access to regular medications and psychosocial interventions was the main factor to psychotic relapse. This review included one cross-sectional clinical study comparing the impact of this pandemic on patients suffering from severe mental illness compared with healthy controls and they found that patients with mental disorders reacted to the pandemic and the lockdown restrictions with higher anxiety levels than the general public. Our study also revealed that elderly people suffering from psychosis and other chronic illness were the most vulnerable to relapse.

**Conclusions:**

Psychotic disorders can relapse during stressful events like COVID-19 pandemic. Therefore, specific attention to these vulnerable subjects is crucial to prevent relapses in times of worldwide pandemic.

**Disclosure:**

No significant relationships.

